# Unveiling the mystery: a rare case of localized malignant pericardial mesothelioma—case report

**DOI:** 10.3389/fonc.2024.1342748

**Published:** 2024-02-26

**Authors:** Yan Zhang, Mingjie Pang

**Affiliations:** ^1^ Department of Magnetic Resonance, The First People's Hospital of Yunnan Province, The Affiliated Hospital of Kunming University of Science and Technology, Kunming, Yunnan, China; ^2^ Department of Cardiology, The First People's Hospital of Yunnan Province, The Affiliated Hospital of Kunming University of Science and Technology, Kunming, Yunnan, China

**Keywords:** 24 h pericardial effusion drainage, wax tissue, late gadolinium enhancement cardiac magnetic resonance, localized malignant pericardial mesothelioma, PET-CT examination

## Abstract

Primary malignant pericardial mesothelioma (PMPM) is a rare pericardial malignant tumor. Most manifestations of PMPM are localized or diffuse masses surrounding the heart. The prognosis of diffuse PMPM is poor due to the difficulty of surgical resection. Although the edge of localized PMPM is clear and can be easily resected, the diagnosis of this disease is difficult. Timely diagnosis and proper treatment are key to a good prognosis. Here, we report a patient with localized PMPM and describe the method for the diagnosis of this disease.

## 1 Introduction

Primary malignant pericardial mesothelioma (PMPM) is a rare pericardial malignant tumor (1, 2). Most PMPM was found in autopsy, and the incidence rate was about 0.006%/0.0022% (3, 4). Malignant pericardial mesothelioma is clinically rare with a poor prognosis and an average survival time of 6–10 months (2). PMPM has not been reported locally. In this study, We present a rare case report of PMPM.

## 2 Case report

A 53-year-old female patient was admitted to our hospital for 6 months because of pericardial effusions. The patient had previous hypertension, with the highest blood pressure of 160/90 mmHg. She had no history of surgery or familial diseases. In the past, the patient had not experienced limb joint pain, morning stiffness, photosensitivity, oral ulcers, hair loss, low-grade fever, and night sweats. She had no weight loss in the short term or recent infections. Physical examination during admission showed stable vital signs. The patient had no obvious symptoms, such as palpitations or chest tightness. On February 10, 2021, the patient underwent Color Doppler Echocardiography in a local hospital for physical examination, which revealed a pericardial effusion. The thickest part was about 4.8 cm, and the pericardium of the left ventricular free wall was thickened and irritable. A CT scan of the chest and abdomen, which was conducted in the local hospital, showed pericardial effusions and calcification in the right lung. Abdominal ultrasound showed no abnormality. Breast ultrasound revealed bilateral breast hyperplasia. Thyroid ultrasound detected TI-RADS3 type of cystic-solid nodules in the right lobe of the thyroid. Suspected pulmonary tuberculosis infection was assessed by local hospitals, and standardized pulmonary tuberculosis chemotherapy was administered. Echocardiography was performed on March 29 after a month of treatment, which showed that the amount of pericardial effusions significantly increased. The thickest part was about 4.8 cm, and the pericardium was localized, thickened, and irritable. The patient visited our hospital on June 21. In addition to the patient’s information stated above, physical examination performed by a specialist showed an enlarged cardiac silhouette, a HR of 83, and a low heart sound. No pericardial friction sounds or abnormal heart sounds were detected.

**Figure 1:**
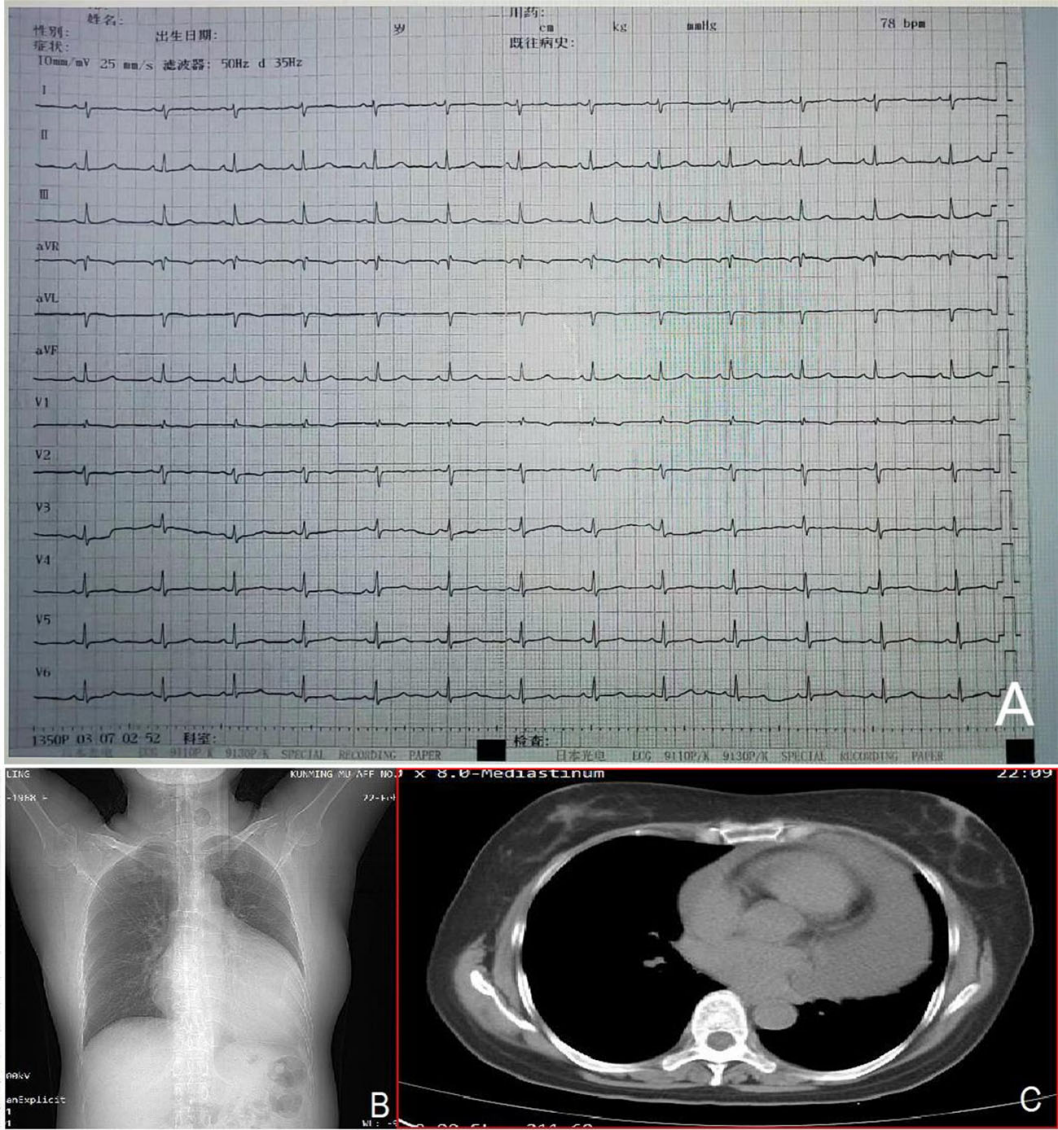
Admission electrocardiogram **(A)** showing a sinus rhythm with low voltage in the chest leads and right bundle branch block. Chest X-ray **(B)** indicating an enlarged heart shadow. Chest CT **(C)** suggesting a large volume of effusions in the pericardial cavity.

**Figure 2:**
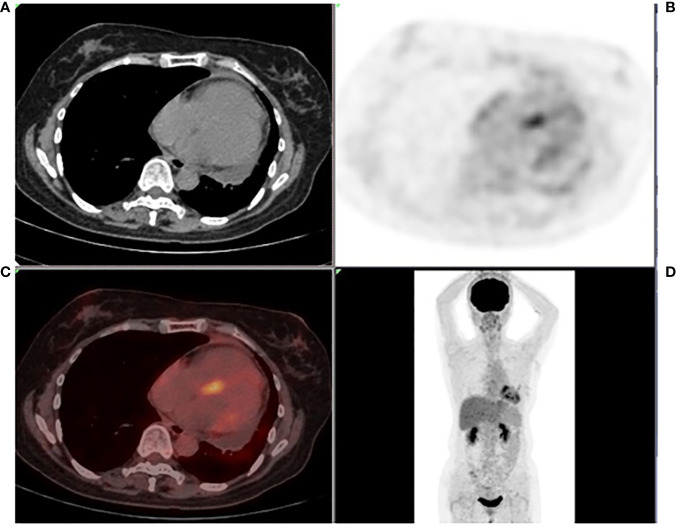
Systemic PET-CT **(A–D)** showing no clear signs of malignant tumor. The pericardium was slightly thickened and adhered, the metabolism increased slightly, and a small amount of effusions were observed in the pericardium with encapsulated changes.

**Figure 3:**
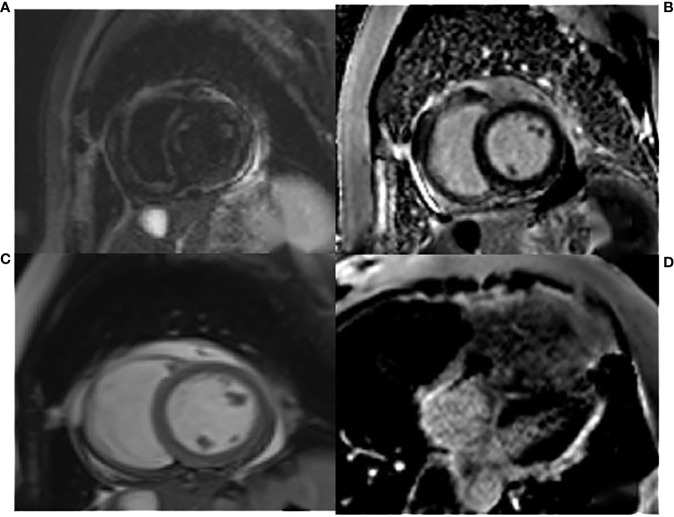
The T2WI fat suppression sequence in **(A)** showed pericardial thickening, adhesive, and lesions on the right ventricular margin adhering to the pleura. The cardiac movie sequence in **(C)** revealed that the fibrous pericardium of the right ventricular free wall adhered to the pleura, the serous pericardium was slightly thickened, and a small amount of pericardial effusions were observed. The LGE images of the two-chamber and four-chamber heart in **(B, D)** indicated thickening and enhancement of the fibrous pericardium.

**Figure 4:**
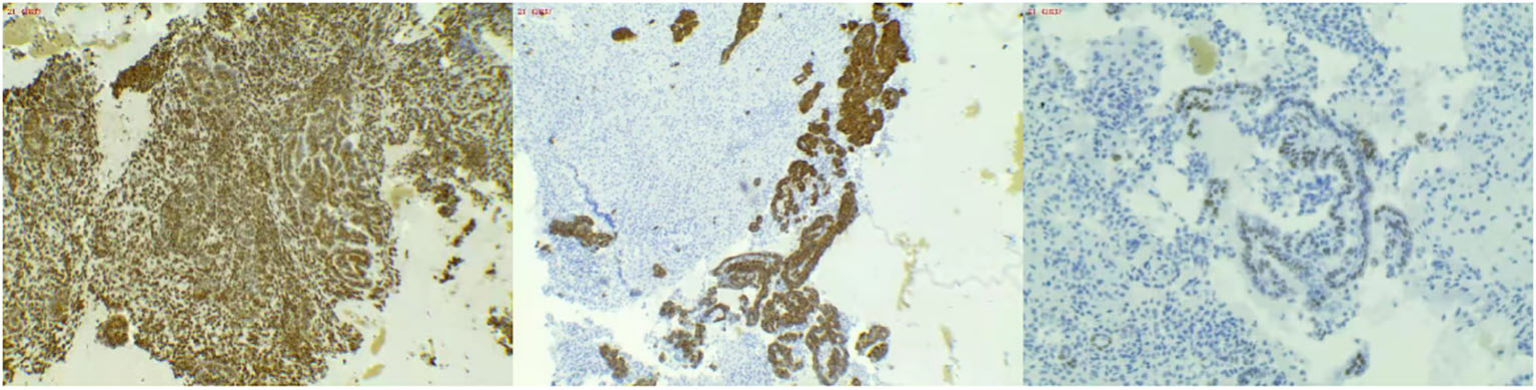
(Wax mass of pericardial effusion) HE morphology and immunohistochemical results consistent with malignant mesothelioma.

The admission electrocardiogram showed a sinus rhythm with low voltage in the chest leads and right bundle branch block (**Figure 1**). Chest X-ray revealed an enlargement of the heart shadow, and chest CT indicated a large volume of pericardial effusions. The blood routine, ESR, and CRP of the patient were normal. No abnormalities were observed in liver and renal functions. The levels of troponin T and Pro-BNP, as well as tumor markers, were normal. Blood ANCA, ANA, RF, and other immune-related indexes were also normal. Blood EB and cytomegalovirus detection revealed no abnormalities. The detection of tuberculosis T cell, tuberculosis antibody, rpoB gene, and mutation of *Mycobacterium* nucleic acid also showed no abnormalities. Pericardial puncture was performed, and fluid was collected simultaneously to improve the relevant laboratory examination. The pericardial effusions were yellow and slightly muddy. The serous mucin qualitative test was positive (+), and the total number of cells was 68 × 10 ^ 6/L. No abnormalities were found in LDH, TP, ADA, GLU, CI, and CEA. Pathological examination of pericardial effusions showed that some of the nuclei were large and deeply stained with a large number of red blood cells and a small number of atypical cells. Perfect PET-CT examination revealed no signs of malignant tumor but showed a slight pericardial thickening and adhesive and a small amount of pericardial effusions with encapsulated changes (**Figure 2**). Then, the late gadolinium enhancement cardiac magnetic resonance examination on the patient showed improvements, such as pericardial thickening, adhesive, lesions on the right ventricular margin, and pleural adhesion. The pericardial fat on the left ventricular free wall appeared blurred, and the fibrous pericardium on the left ventricular free wall exhibited thickening. Perfusion imaging indicated that the thickened hairy pericardium had moderate enhancement. The late gadolinium contrast agent enhancement (LGE) medium implied that the pericardial thickening exhibited delayed enhancement (**Figure 3**). During hospitalization, the second pericardiocentesis was performed, pericardial effusion was drained for 24 h, and the sediment was collected for wax-encapsulated pathological examination and immunohistochemistry. Immunohistochemical staining showed the following expression of tumor cells: CK7(+), CK20(−), Villin(−), TTF-1(−), WT-1(+), P16(+), CR(+), CDX-2(−), CA125(+), Pax-8(−), P53(+), CK19(+), CK8(+), CK5/6(+), Vim(+), ER(−), PR(−), and Ki-67(+). Collectively, these results suggested malignant mesothelioma (**Figure 4**). The patient was then transferred to the department of oncology, but the patient refused chemotherapy. For nearly a year and a half, the patient was followed up regularly. As of September 30, 2023, the patient had stable vital signs and had no metastasis in other body parts.

## 3 Discussion

Pericardial tumors are rare in clinical works, and most of them come from adjacent tissues and organs, such as malignant pleural tumors, malignant lung tumors, melanomas, or lymphomas. Primary pericardial malignant tumors are rare. The prevalence of PMPM in pericardial tumors is less than 0.002% (2). The cause of PMPM is also unknown. Notably, malignant pleural mesothelioma is usually associated with asbestos exposure (5), but patients mentioned in previous literature had no history of asbestos exposure and no obvious etiology (6). No particular age of onset was reported. Clinical symptoms in previous reports were not characteristic and regular, and the typical manifestation is the clinical presentation of pericardial effusions (6).

The prognosis of PMPM is poor, the survival time is short, and the reported survival time is about 2.5 years (7). A correct clinical diagnosis needs to be made as soon as possible. The lack of characteristic clinical manifestations of PMPM causes great challenges to the diagnosis of the disease. Thus, improving imaging and pathological examination is key to its diagnosis and differentiation, especially in cases of localized pericardial thickening. Imaging examination methods include cardiac color Doppler ultrasound, CT, PET-CT, and CMR. Color Doppler echocardiography and CT can detect pericardial effusions, pericardial thickening, or localized pericardial masses. Enhanced CT can also indicate the blood supply of tumors. PET-CT not only can detect pericardial effusions and masses but also can identify or exclude other tumors. In the current case, the pericardial focus of the patient is limited and flocculent, not a formed mass, which causes difficulties in diagnosis. CMR has high soft tissue resolution and can be used for multi-parameter and multi-directional imaging. CMR can dynamically observe the relationship between cardiac movement and the pericardium. It can identify the delayed enhancement of the pericardium after thickening on LGE sequences, which is valuable in the localization and qualitative diagnosis of diseases.

In this case, the focus of the patient is limited, and the positive rate of pericardial effusion is low. A special way to collect pericardial effusions is necessary. The conventional method of retaining pericardial effusions involves collecting fluid immediately after puncture for laboratory examination and pathological examination. In this case, 24-hour pleural effusion was drained and filtered, and the sediment was collected and wrapped in wax blocks.

The sediment wrapped in wax blocks was investigated by pathological examination and immunohistochemistry. The use of wax tissue avoids a low positive rate of diagnosis and missed diagnosis caused by a small number of cells, overlapping accumulation of cells, uneven smear thickness, and multiple background impurities. Wax block tissue has various advantages, such as ease of acquisition, low cost, simple operation, and good specificity. At present, cell wax block diagnosis is the most effective and reliable technique to distinguish benign and malignant effusions. The combination of immunocytochemical staining can help in identifying the primary source, identify the pathological type of the tumor, and further conduct molecular pathological detection, which provides an objective basis for accurate treatment and prognosis of patients and is valuable for extensive clinical promotion (8, 9). In summary, localized pericardial thickening and massive pericardial effusions are easily missed and misdiagnosed in patients. At the same time, the positive rate of pleural effusion is low, which cannot be used as a diagnostic criterion of negative PMPM (10, 11). For such cases, various laboratory tests and imaging methods need to be improved, and special methods should be adopted for pleural effusion drainage and pathological examination. Best efforts should be made to avoid the progression of diseases and increase the survival rate of patients.

## Data availability statement

The original contributions presented in the study are included in the article/supplementary material. Further inquiries can be directed to the corresponding author.

## Ethics statement

The studies involving humans were approved by Ethics Committee of Yunnan First People’s Hospital. The studies were conducted in accordance with the local legislation and institutional requirements. The participants provided their written informed consent to participate in this study. Written informed consent was obtained from the individual(s) for the publication of any potentially identifiable images or data included in this article.

## Author contributions

YZ: Writing – original draft. MP: Writing – review & editing.
